# Antitumor Activity of Liposomal Nanoparticles Co-Encapsulating Ceramides and Doxorubicin in In Vitro Nucleolin-Expressing Neuroblastoma Models

**DOI:** 10.3390/cells15110958

**Published:** 2026-05-22

**Authors:** Veronica Bensa, Hugo Lopes-Cardoso, Martina Ardito, Eleonora Ciampi, Anastasiya Voronovska, João Soares-Gonçalves, Mirco Ponzoni, Chiara Brignole, João Nuno Moreira, Fabio Pastorino

**Affiliations:** 1 Laboratory of Experimental Therapies in Oncology, IRCCS Istituto Giannina Gaslini, 16147 Genoa, Italy; veronicabensa@gaslini.org (V.B.); martinaardito@gaslini.org (M.A.); eleonoraciampi@gaslini.org (E.C.); mircoponzoni@gaslini.org (M.P.); chiarabrignole@gaslini.org (C.B.); 2 CNC-UC-Center for Neurosciences and Cell Biology, Center for Innovative Biomedicine and Biotechnology (CIBB), University of Coimbra, Rua Larga—Faculdade de Medicina, 1ºandar—POLO I, 3004-504 Coimbra, Portugal; hugoeb2013@gmail.com (H.L.-C.); anastasiavoron98@gmail.com (A.V.); joaogoncalvesskmr@gmail.com (J.S.-G.); jmoreira@ff.uc.pt (J.N.M.); 3 CIBB, Polo das Ciências da Saúde, University of Coimbra, Azinhaga de Santa Comba, 3000-548 Coimbra, Portugal

**Keywords:** neuroblastoma, nucleolin, drug delivery, liposomes

## Abstract

**Background:** Neuroblastoma (NB) causes about 15% of cancer deaths in childhood. Recently, we suggested cell-surface nucleolin (NCL) as a novel target for preclinical therapy against NB. **Methods:** Here, a broad range of human NB cell lines were evaluated for NCL expression. PEGylated liposomal nanoparticles, co-encapsulating C6- or C18-ceramides and doxorubicin (DXR) and functionalized with the F3 peptide (F3-lipo[C6-DXR] or F3-lipo[C18-DXR]), were tested against NCL-expressing NB cell lines, grown in monolayers (2D) and as multicellular tumor spheroids (3D). Untargeted liposomes were used as the control. Cytotoxicity and apoptotic/necrotic deaths were evaluated. **Results:** All NB cell lines expressed cell-surface NCL. Compared to untargeted formulations, F3-lipo[C6-DXR] and F3-lipo[C18-DXR] showed enhanced cellular association and antitumor effects against NB cells. Compared to F3-lipo[C18-DXR], F3-lipo[C6-DXR] was significantly more effective in reducing 2D and 3D NB cell lines’ viability (2D: IC50 range 313–995 nM and 239–629 nM, respectively; 3D: IC50 range 202–416.2 nM and 62.61–398.6 nM, respectively) and in inducing apoptotic cell death. F3-lipo[C6-DXR] also led to a greater cytotoxicity compared to liposomal DXR alone, highlighting the benefit of co-encapsulation. **Conclusions:** NCL is a promising target in NB, and F3-targeted liposomes enable the selective delivery of their cargo. F3-lipo[C6-DXR] showed superior antitumor activity, supporting ceramide–DXR co-encapsulation as a potential treatment strategy, which needs to be further validated.

## 1. Introduction

Cancer is still the second leading cause of mortality worldwide, with millions of new diagnoses every year. However, due to early diagnosis and innovative therapies, the mortality rate is decreasing [1]. Neuroblastoma (NB), a pediatric tumor that originates during the development of the sympathetic nervous system, causing about 15% of cancer deaths in childhood, remains difficult to treat, especially in patients with the high-risk (HR) form of the disease [2]. NB is characterized by a broad heterogeneity in terms of biological and clinical features. This has led to the need to cluster patients into risk groups to better guide treatment approaches. Patients are classified into very-low-risk (VLR), low-risk (LR), intermediate-risk (IR), and HR groups. This stratification also reflects the prognosis of patients, which, despite intensive treatment regimens, including high-dose chemotherapy, radiotherapy, and immunotherapy, continues to be poorer in HR patients, with an overall survival rate of 50% [2,3]. Moreover, the survival rate of relapsed, HR NB patients is below 20%. Current multimodal therapies lack specific cellular targets, thus causing systemic toxicity to healthy organs and the occurrence of drug resistance. In this regard, nanomedicine is the application of nanotechnology to healthcare for the prevention, diagnosis, monitoring, and treatment of disease. Specifically, the use of nanocarriers (mainly liposomes or nanoparticles) to deliver drugs and molecules (i.e., chemotherapeutics, nucleic acids, and small molecules) to diseased tissues enhances their pharmacokinetic profiles, preventing rapid clearance and improving bioavailability [4]. Nanocarriers take advantage of the enhanced permeability and retention (EPR) effect, according to which the leaky tumor vasculature and the deficient tumor lymphatic system allow their passive penetration and accumulation into tumors [5]. Moreover, nanocarriers can be functionalized with moieties, such as antibodies or peptides [6], in order to increase tumor targeting, while sparing normal tissues. Liposomes are among the most widely studied and clinically employed nanocarriers due to their characteristics of biocompatibility and bioavailability. They can be loaded with both hydrophilic and hydrophobic compounds, making possible the simultaneous delivery of synergistic drug combinations that can enhance the efficacy of single-agent therapies and circumvent drug resistance [6,7,8]. In this scenario, ceramides have attracted a lot of interest as antitumor molecules, as they can modulate cancer-associated pathways [9,10]. Indeed, ceramides are sphingolipids physiologically involved in the regulation of cellular processes including apoptosis, proliferation, cell differentiation, and metabolism [11]. In cancer cells, ceramide levels are deregulated, contributing to cancer progression. For this reason, the delivery of ceramides has been explored as a new approach for cancer therapy [11,12]. Sphingolipids are composed of three main elements: a sphingoid base, a fatty acid chain, and a head group. Specifically, ceramides consist of a sphingosine backbone linked to a fatty acid through an amide bond and are characterized by carbon chains of differing lengths [13]. Previous studies have shown that the use of ceramide analogs can enhance the levels of ceramides inside tumor cells, and their administration in combination with traditional chemotherapeutic drugs, such as doxorubicin, cisplatin, and tamoxifen, enhances apoptosis induction in cancer cells [14]. Their effectiveness has also been widely tested in various cancer models [15]. Because of the hydrophobic nature of ceramides, the use of short-chain fatty acids ceramide analogs and their encapsulation into nanocarriers can maximize their pharmacological effects [16]. Different types of ceramides display different activities and properties. Both short- and long-chain ceramides have been tested in vitro and in vivo in several preclinical cancer models. Short-chain ceramide analogs improve ceramide bioavailability, while long-chain ceramides better resemble the natural ceramides of cell membranes [11]. Among the short-chain ceramides, C6-ceramide is the most widely studied [17].

Recently, we suggested cell-surface nucleolin (NCL) as a novel target for preclinical therapy against NB [18,19]. In this study, PEGylated liposomal nanoparticles, co-encapsulating C6- or C18-ceramides and doxorubicin (DXR) and functionalized with the F3, NCL-recognizing peptide (F3-lipo[C6-DXR], or F3-lipo[C18-DXR]), were tested against NCL-expressing NB cell lines, grown in monolayers (2D) and as multicellular tumor spheroids (3D). The antitumor activity obtained in vitro supports ceramide–DXR co-encapsulation as a potential treatment strategy to be further validated in clinically relevant animal models of NB.

## 2. Materials and Methods

### 2.1. Human Cell Cultures

The human IMR-32, SH-SY5Y, SK-N-AS, HTLA-230 and SK-N-BE(2)-C neuroblastoma (NB) cell lines were cultured in DMEM HIGH Glucose medium, supplemented with 10% FBS, 1% penicillin/streptomycin, and 1% L-glutamine. The human ACN, GI-ME-N, NB-1691 and SHEP-21N NB cell lines and the human keratinocyte cell line HaCaT, used as a healthy control, were cultured in RPMI-1640 medium, supplemented with 10% FBS, 1% non-essential amino acids, 2% HEPES, 1% penicillin/streptomycin, and 1% L-glutamine.

### 2.2. Nucleolin Expression Evaluation

Cell-surface nucleolin (NCL) expression was evaluated by means of flow cytometry (FC) analysis (Becton-Dickinson Immunocytometry Systems, Gallios Flow Cytometer, Beckman Coulter, Brea, CA, USA) on NB cell lines and on the human keratinocyte cell line HaCaT. Specifically, 2 × 10^5^ NB or healthy cells were incubated for 25 min at 4 °C with an anti-NCL (mouse IgG1 moAb, AlexaFluor488, clone 364–5, Abcam, Cambridge, UK) monoclonal antibody (moAb). After washing with 2 mM EDTA and 1% FBS, cells were analyzed via FC. NCL expression was reported as the mean ratio fluorescence intensity (MRFI) over unlabeled control cells ± standard deviation (S.D.).

### 2.3. Development of NCL-Recognizing Liposomal Nanoparticles Co-Encapsulating Ceramides and Doxorubicin

Non-targeted pH-sensitive liposomes were composed of DOPE:CHEMS:DSPC:Cholesterol:DSPE-PEG_2k_:C6- or C18-ceramide at a molar ratio of 24.6:12.4:18.5:18.5:7.4:18.5, whereas targeted pH-sensitive liposomes were composed of DOPE:CHEMS:DSPC:Cholesterol:DSPE-PEG_2k_:DSPE-PEG-F3:C6- or C18-ceramide at a molar ratio of 24.6:12.4:18.5:18.5:5.7:1.7:18.5. Liposomes were prepared by means of the ethanol injection method, as previously described [12]. Drug loading was achieved by incubating pre-formed liposomes with doxorubicin (DXR) at a drug-to-total lipid molar ratio of 0.18:1 (corresponding approximately to a ceramide:DXR molar ratio of 1:1) for 60 min at 60 °C. Non-encapsulated drugs were removed using a Sephadex G-50 gel column (Merck, Darmstadt, Germany)equilibrated with 25 mM HEPES and 10% (*m*/*v*) sucrose buffer (pH 7.4). The z-average, the polydispersity index (PDI), and the zeta-potential of the liposomes were determined by means of dynamic light scattering (DLS) using a Zetasizer Ultra Red (Malvern Panalytical, Malvern, UK).

Targeted liposomes were obtained by incorporating DSPE-PEG_2k_-F3 directly into the ethanolic lipid mixture, as previously reported [19].

### 2.4. Cellular Association and Uptake of NCL-Recognizing Liposomal Nanoparticles Co-Encapsulating Ceramides and Doxorubicin

From here onward, the untargeted liposomal formulations co-encapsulating ceramides and doxorubicin will be referred to as lipo[C6-DXR] and lipo[C18-DXR], while the NCL-targeted formulations will be referred to as F3-lipo[C6-DXR] and F3-lipo[C18-DXR]. The uptake behavior of the F3-functionalized formulations has already been characterized in NB cell lines and reproduced in our previous studies using conventional approaches such as flow cytometry and confocal microscopy [18]. Therefore, the cellular association and internalization of lipo[C6-DXR], lipo[C18-DXR], F3-lipo[C6-DXR], and F3-lipo[C18-DXR] were evaluated here in a single confirmatory proof-of-concept experiment performed on two NB cell lines via real-time timelapse acquisition using a ptychography-based instrument (Livecyte, Phasefocus, Sheffield, UK). Specifically, IMR-32 and SH-SY5Y cell lines were seeded in glass-bottomed 8-well chamber slides (2 × 10^4^ cells per well). The day after seeding, cells were incubated with the different liposomal formulations (0.25 μM DXR) and timelapse acquisition of the red fluorescence emitted by DXR was carried out for up to 10 h, with a scan every 30 min.

### 2.5. Antitumor Effectiveness of NCL-Recognizing Liposomal Nanoparticles Co-Encapsulating Ceramides and Doxorubicin Against NCL-Expressing NB Cell Lines

For 2D cultures, NB cells were seeded in different amounts, depending on the cell line (5.8 × 10^3^–9.6 × 10^3^ per well), in 96-well plates and, the day after, treated for 30 min at 37 °C with untargeted (lipo[C6-DXR] and lipo[C18-DXR]) and NCL-recognizing (F3-lipo[C6-DXR] and F3-lipo[C18-DXR]) liposomal formulations. The liposomal formulations were administered based on their DXR concentration, and increasing doses of doxorubicin (DXR) (0.25–2 µM) were used. After 72 h of treatment, the cell viability was determined by the use of an MTS tetrazolium salt (3-(4,5-dimethylthiazol-2-yl)-5-(3-carboxymethoxyphenyl)-2-(4-sulfophenyl)-2H-tetrazolium) assay according to supplier’s instructions (CellTiter 96^®^ AQueous One Solution Cell Proliferation Assay (Promega, Milan, Italy), by recording absorbance at 490 nm using a microplate reader (Glomax, Promega). The viability of 2D models was expressed as a percentage (%) of viability over the control (CTR) ± standard deviation (S.D.).

For 3D cultures, 2.8 × 10^3^ NB cells per well were seeded in 96-well ultra-low attachment plates. Three days after seeding, the period required for spheroid formation, 3D cultures were treated for 2 h at 37 °C with F3-lipo[C6-DXR] and F3-lipo[C18-DXR] (0.5–2 μM DXR). Cell viability was determined 4 days after treatment using the CellTiter-Glo^®^ 3D Cell Viability kit. The viability of 3D models was expressed as a percentage (%) of viability over the control (CTR) ± standard deviation (S.D.).

In separate experiments, the cytotoxic activity of F3-lipo[C6-DXR] was compared with that of liposomal DXR alone (F3-lipo[DXR]) against both 2D and 3D NB models. The viability of treated and control samples was assessed as described above.

### 2.6. In Vitro Apoptosis/Necrosis Death Assay

IMR-32 and SK-N-AS NB cells were seeded in 96-well plates (8.7 × 10^3^ cells/well and 5.8 × 10^3^ cells/well, respectively) and treated, as reported above, with 0.25–0.5 µM DXR loaded in either F3-lipo[C6-DXR] or F3-lipo[C18-DXR]. The RealTime-Glo™ Annexin V Apoptosis and Necrosis Assay (Promega) was used, according to the manufacturer’s instructions. Results were recorded through the GloMax Discover instrument.

### 2.7. Statistics

In vitro cytotoxicity experiments were performed at least five times, with an average of four technical replicates for each treatment condition intraexperiment. Apoptosis/necrosis assays were performed four times. IC_50_ values were obtained by means of nonlinear curve fitting using the sigmoidal dose–response (variable slope) equation in GraphPad Prism 5. Statistical analyses were performed with Prism 5 software (GraphPad, La Jolla, CA, USA): one-way analyses of variance (ANOVA) with Tukey’s Multiple Comparison Test and unpaired *t*-test, one-tailed, were used to evaluate differences within treatments. Asterisks indicate the following *p*-value ranges: * = *p* < 0.05, ** = *p* < 0.01, *** = *p* < 0.001.

## 3. Results

### 3.1. Cell-Surface Nucleolin Is Expressed on Neuroblastoma Tumor Cell Lines

**Figure 1:**
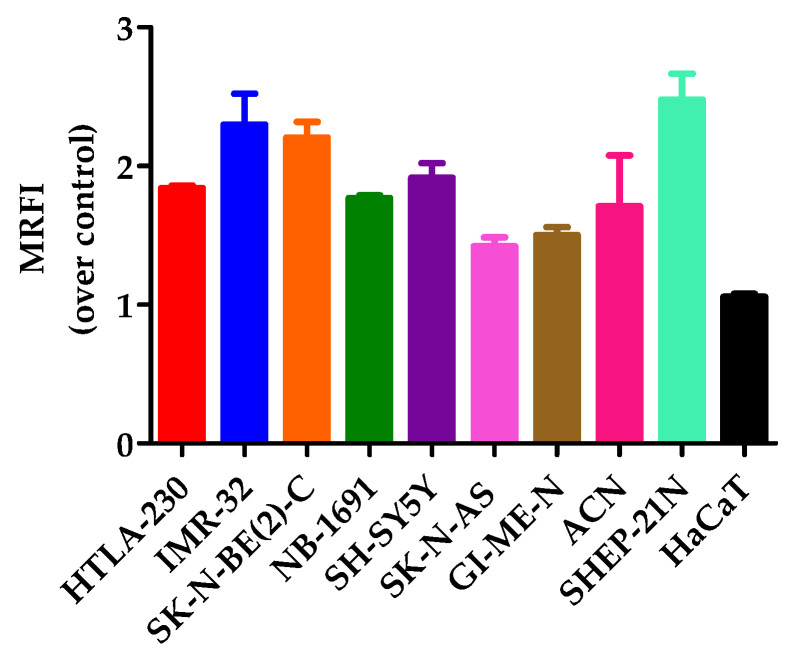
Flow cytometry analysis of the expression of cell-surface nucleolin on neuroblastoma cell lines. Human NB (adrenergic-type: HTLA-230, IMR-32, SK-N-BE(2)-C, NB-1691 and SH-SY5Y; mesenchymal-type: SK-N-AS, GI-ME-N, ACN and SHEP-21N) cell lines and human keratinocytes (HaCaT) were stained with anti-NCL AlexaFluor488. NCL expression was evaluated via flow cytometry. NCL expression is reported as mean relative fluorescence intensity (MRFI) normalized over the control unstained cells. Columns: MRFI ± standard deviation (S.D.).

Constitutive expression of cell-surface nucleolin (NCL) was evaluated in a broad range of human neuroblastoma (NB) cell lines by means of flow cytometry (FC) analysis. Human skin keratinocytes were used as controls. Confirming previous findings [18], FC analysis demonstrates that NCL was expressed, to differing extents, on the cell surface of all adrenergic-type and mesenchymal-type NB cell lines analyzed, while its expression on healthy cells was negligible (Figure 1), indicating that cell-surface NCL expression is tumor-specific.

### 3.2. Nucleolin-Recognizing Liposomal Nanoparticles Co-Encapsulating Ceramides and Doxorubicin Were Associated with Neuroblastoma Cells Exerting Specific Antitumor Efficacy

Non-targeted and NCL-targeted liposomes co-encapsulating ceramides (C6 or C18) and doxorubicin, whose physicochemical features are reported in Table 1, were first tested for their association with NB cell lines. The DXR-derived fluorescence associated with the cells after incubation served as a marker of cellular binding/uptake.

**Figure 2:**
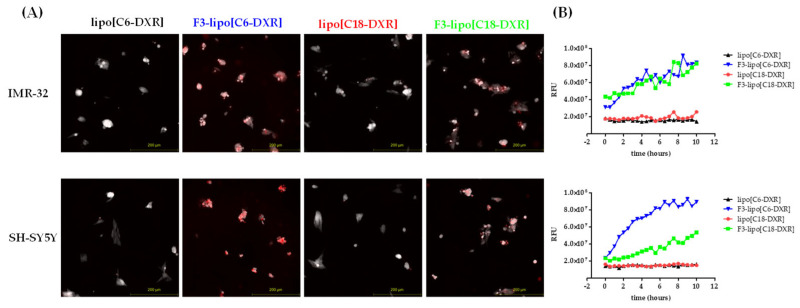
Cellular association of co-encapsulated liposomal nanoparticles of ceramides and doxorubicin on neuroblastoma cells. (**A**) Cellular association efficiency of F3-targeted and untargeted liposomes on IMR-32 and SH-SY5Y neuroblastoma cell lines. Red: DXR-derived fluorescence. (**B**) Fluorescence quantification of cellular association efficiency of F3-targeted and untargeted liposomes on IMR-32 (upper) and SH-SY5Y (bottom). Black: lipo[C6-DXR]; blue: F3-lipo[C6-DXR]; red: lipo[C18-DXR]; green: F3-lipo[C18-DXR].

Compared to the non-targeted formulations, F3-lipo[C6-DXR] and F3-lipo[C18-DXR] showed an enhanced time-dependent cellular association (Figure 2).

**Figure 3:**
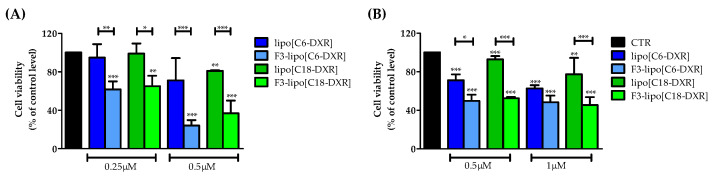
Antitumor effect of co-encapsulated liposomal nanoparticles of ceramides and doxorubicin on neuroblastoma cells cultured as monolayers. Cytotoxicity induced by untargeted (lipo[C6-DXR] and lipo[C18-DXR]) and NCL-recognizing (F3-lipo[C6-DXR] and F3-lipo[C18-DXR]) liposomes on IMR-32 (**A**) and SH-SY5Y (**B**) neuroblastoma cell lines. Histograms represent the percentage (%) of cell viability over the control (CTR) ± standard deviation (S.D). 0.5–2 μM: Doses of DXR used. Statistics: One-way analyses of variance (ANOVA) with Tukey’s Multiple Comparison Test, *, *p* < 0.05; **, *p* < 0.01; ***, *p* < 0.001.

On the same cell lines, we subsequently evaluated whether the increased cellular association of the nanoparticles functionalized with the F3 peptide, which enables us to recognize cell-surface NCL, translated into an increase in their cytotoxic effect. As expected, the F3-targeted formulations led to a significant cell viability reduction compared to the respective untargeted ones (Figure 3).

The superior effect of the F3-targeted formulations was subsequently also confirmed on the SK-N-AS mesenchymal-type cell line (Figure S1); however, this cell line was found to be less treatment-sensitive compared to the adrenergic-type cell lines.

**Figure 4:**
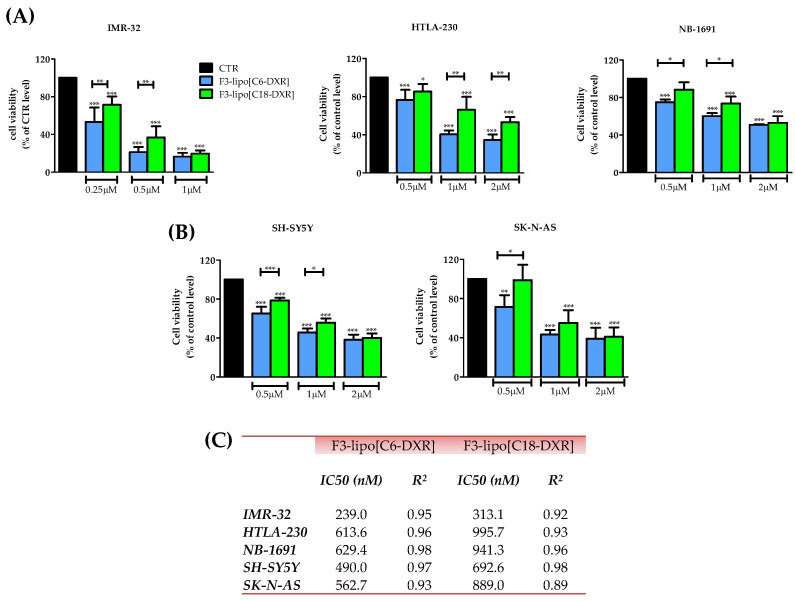
Antitumor effect of NCL-recognizing co-encapsulated liposomal nanoparticles of ceramides and doxorubicin on neuroblastoma cells cultured as monolayers. Cytotoxicity induced by NCL-recognizing (F3-lipo[C6-DXR] and F3-lipo[C18-DXR]) liposomes on *MYCN*-amplified (**A**) and *MYCN* single-copy (**B**) neuroblastoma cell lines. Histograms represent the percentage (%) of cell viability over the control (CTR) ± standard deviation (S.D). 0.25–2 μM: Doses of DXR used. Statistics: One-way analyses of variance (ANOVA) with Tukey’s Multiple Comparison Test, *, *p* < 0.05; **, *p* < 0.01; ***, *p* < 0.001. (**C**) IC50 values calculated for F3-lipo[C6-DXR] and F3-lipo[C18-DXR] formulations.

Based on the results obtained, the study continued by exclusively testing the liposomal nanoparticles functionalized with the F3 peptide. Indeed, a specific comparison, in terms of the cytotoxic effect, was carried out by comparing the two formulations targeting NCL. The experiments were performed on a panel of NB cell lines reflecting the tumor heterogeneity typical of NB. Specifically, the cell viability experiments were performed on five NB cell lines grown in monolayers (2D). Cells were incubated with F3-lipo[C6-DXR] and F3-lipo[C18-DXR] nanoparticles, which were administered, based on their payload concentration, with increasing doses of DXR, in order to calculate their IC50 values. As shown in Figure 4, all the analyzed NB cells were sensitive to both NCL-recognizing liposomal formulations, although to a different extent and in a dose-dependent manner. Notably, F3-lipo[C6-DXR] was more effective in reducing the number of viable cells compared to F3-lipo[C18-DXR] for both *MYCN*-amplified and *MYCN*-single copy NB cell lines (Figure 4A,B). Statistically significant differences were observed for specific doses used. Even more importantly, the calculated IC50 values were always higher for F3-lipo[C18-DXR] compared to F3-lipo[C6-DXR] (Figure 4C).

### 3.3. Nucleolin-Recognizing Liposomal Nanoparticles Co-Encapsulating Ceramides and Doxorubicin Induce Apoptotic Cell Death in Neuroblastoma Cells

**Figure 5:**
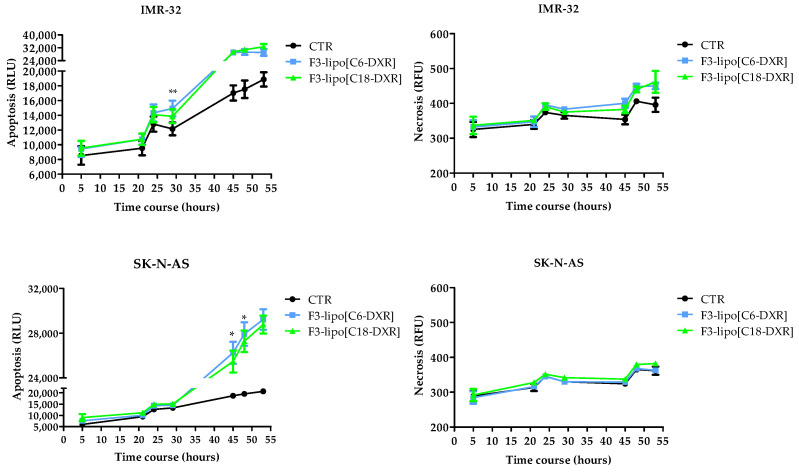
Apoptotic/necrotic cell death evaluation on neuroblastoma cells incubated with NCL-recognizing co-encapsulated liposomal nanoparticles of ceramides and doxorubicin. Kinetics of apoptosis and necrosis, detected in luminescence (RLU) and fluorescence (RFU), respectively, on IMR-32 and SK-N-AS neuroblastoma cell lines. Each dot represents the mean ± standard deviation (S.D). IMR-32 and SK-N-AS cells were treated with 0.25 µM and 0.5 µM of liposomal DXR, respectively. Statistics, F3-lipo[C6-DXR] vs. F3-lipo[C18-DXR]: unpaired *t*-test, one-tailed *p* value. *, *p* < 0.05; **, *p* < 0.01.

The mechanism of cell death induced by the F3-lipo[C6-DXR] and F3-lipo[C18-DXR] formulations was investigated on IMR-32 (adrenergic phenotype) and SK-N-AS (mesenchymal phenotype) cell lines cultured in monolayers. As shown in Figure 5, both the liposomal formulations led to a progressive increase in apoptotic cell death compared to the control untreated cells, while necrosis was almost negligible and superimposable compared to the control. Importantly, at specific time points, a slight, although significant, difference was recorded between F3-lipo[C6-DXR] and F3-lipo[C18-DXR] (i.e., IMR-32: at 29 h; SK-N-AS: at 45 and 48 h).

### 3.4. Nucleolin-Recognizing Liposomal Nanoparticles Co-Encapsulating Ceramides and Doxorubicin Exert Specific Antitumor Efficacy Against Neuroblastoma Cells Cultured as Multicellular Tumor Spheroids

**Figure 6:**
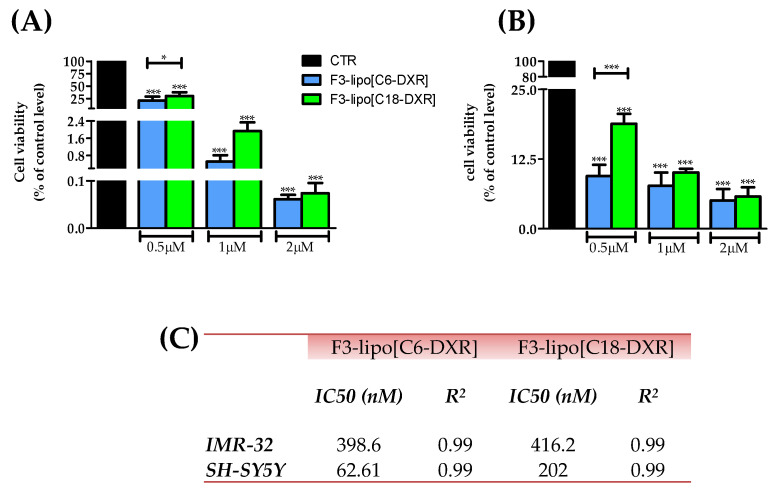
Antitumor effect of NCL-recognizing co-encapsulated liposomal nanoparticles of ceramides and doxorubicin on neuroblastoma cells cultured as multicellular tumor spheroids. Cytotoxicity induced by NCL-recognizing (F3-lipo[C6-DXR] and F3-lipo[C18-DXR]) liposomes on multicellular tumor spheroids (3D) derived from IMR-32 (**A**) and SH-SY5Y (**B**) NB cell lines. Histograms represent the percentage (%) of cell viability of treated cells over untreated (CTR) 2D and 3D cells ± standard deviation (S.D). 0.25–2 μM: Doses of DXR used. Statistics: One-way analyses of variance (ANOVA) with Tukey’s Multiple Comparison Test, *, *p* < 0.05; ***, *p* < 0.001. (**C**) IC50 values calculated for F3-lipo[C6-DXR] and F3-lipo[C18-DXR] formulations.

Multicellular tumor spheroids (3D) represent a three-dimensional tumor-like cellular organization that better reflects the tridimensional structure of tumors observed in patients. Based on this, the antitumor effect of F3-C6-DXR and F3-C18-DXR liposomes was also evaluated on multicellular tumor spheroids derived from IMR-32 and SH-SY5Y NB cell lines. In both models, F3-lipo[C6-DXR] was more effective in reducing the number of viable cells compared to F3-lipo[C18-DXR] (Figure 6A,B). IC50 values further strengthened the superior antitumor efficacy of F3-lipo[C6-DXR] (Figure 6C), reflecting the results obtained for NB cells cultured as a monolayer (Figure 4).

### 3.5. Nucleolin-Recognizing Liposomal Nanoparticles Co-Encapsulating C6-Ceramide and Doxorubicin Exert Superior Antitumor Efficacy Against Neuroblastoma Cells Compared to Liposomal Doxorubicin Alone

**Figure 7:**
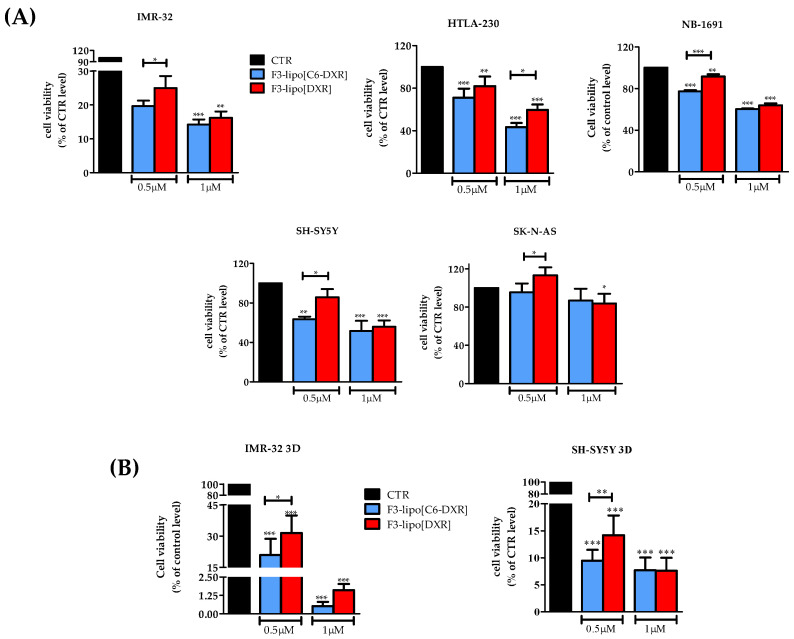
Comparison of the antitumor effect of NCL-recognizing co-encapsulated liposomal nanoparticles of C6-ceramide and doxorubicin and of liposomal doxorubicin alone. Cytotoxicity induced by NCL-recognizing co-encapsulated liposomal nanoparticles of C6-ceramide and DXR liposomes (F3-lipo[C6-DXR]) and liposomal DXR alone (F3-lipo[DXR]) on NB cells cultured as monolayers (2D) (**A**) and as multicellular tumor spheroids (3D) (**B**). Histograms represent the percentage (%) of cell viability of treated cells over untreated (CTR) 2D and 3D cells ± standard deviation (S.D). Statistics: One-way analyses of variance (ANOVA) with Tukey’s Multiple Comparison Test, *, *p* < 0.05; **, *p* < 0.01; ***, *p* < 0.001.

Then, the antitumor activity of F3-lipo[C6-DXR] was compared with the liposomal formulation encapsulating DXR alone (F3-lipo[DXR]), whose antitumor efficacy against NB cell lines and patient-derived cells has previously been reported [18,19]. These experiments were performed on NB cells cultured both in monolayers and as multicellular tumor spheroids. F3-lipo[C6-DXR] was found to be significantly more cytotoxic than liposomal DXR alone, highlighting the benefits of co-encapsulation (Figure 7).

## 4. Discussion

In this study, we demonstrated that liposomal nanoparticles, functionalized with the F3 peptide and co-encapsulating ceramides and doxorubicin (DXR), exerted a potent cytotoxic effect against neuroblastoma (NB) models in vitro. Specifically, nanoparticles co-encapsulating C6-ceramide and DXR show superior antitumor activity compared to nanoparticles co-encapsulating C18-ceramide and DXR and compared to liposomal DXR alone, further supporting the benefits of co-encapsulation. NB is a tumor that arises from cells of the developing sympathetic nervous system and represents the most frequent and aggressive form of extracranial solid tumor in childhood [2,20]. In recent years, current therapies have improved the outcomes of patients with NB, but the lack of specific cellular targets can cause systemic toxicity and drug resistance, resulting in poor outcomes, particularly for high-risk NB patients [2]. For these reasons, innovative NB-targeted therapeutic strategies are urgently needed.

Nucleolin (NCL) is one of the most abundant proteins of the nucleolus, involved in controlling DNA and RNA metabolism, ribosome biogenesis, rRNA synthesis and processing, cell proliferation, angiogenesis, and microRNA processing, among other things [21,22,23]. However, it is also implicated in pathological conditions, especially in tumorigenesis and viral infection, rendering NCL a target for the development of antitumor and antiviral strategies [22,23]. Furthermore, in cancer, NCL is not located only in the nucleolus, but also in the cytoplasm and on the cell membrane, representing a potential target for treatment.

Nanocarriers, such as liposomes, are recognized as useful and biocompatible tools for drug delivery [4]. Moreover, tumor-targeting moieties (i.e., antibodies, aptamers, and/or peptides) can specifically guide therapeutic nanoparticles toward the tumor tissues, while sparing healthy organs [4]. As already described, the F3 peptide has the ability to recognize cell-surface NCL protein. Based on our previous findings [12,18,19], in this study the specificity of the targeting and the effectiveness of F3-targeted liposomal formulations were confirmed on a broad panel of human NB cell lines, grown in monolayers (2D) and as multicellular tumor spheroids (3D), through cell association and cytotoxicity experiments. Through timelapse single-cell segmentation analysis, we demonstrated that F3-liposomes encapsulating ceramides and DXR were able to specifically associate to NCL-expressing tumor cells in a faster and more efficient manner than the non-targeted formulations. This ultimately resulted in a more pronounced reduction in NB cell viability.

Ceramides are known to be key mediators of antitumor pathways, as they are involved in the regulation of cellular processes such as apoptosis and proliferation, and low levels of ceramides are detected in several types of tumors [11]. These findings support their use in association with chemotherapeutics in order to improve treatment outcomes and overcome drug resistance. In previous studies, the synergistic action of conventional chemotherapy in association to ceramides, delivered through liposomes, has been reported in in vitro and in vivo preclinical tumor models [24,25,26,27]. Specifically, Chen L. and Cordeiro Pedrosa L.R. demonstrated that liposomes co-encapsulating DXR and ceramides exerted enhanced cytotoxic effects, compared to liposomal DXR alone, against melanoma, breast carcinoma, and pancreatic carcinomas cell lines in vitro [24,25]. Zhai L. and colleagues demonstrated that liposomal short-chain C6-ceramide induces potent anti-osteosarcoma effect in vitro and in vivo, sensitizing the activity of methotrexate and doxorubicin [26]. Recently, we also observed the enhanced antitumor effect of targeted liposomal doxorubicin/ceramides combinations in in vitro models of breast and ovarian cancer, highlighting the superior cytotoxic activity exerted by C6-ceramide compared to liposomal DXR alone [27].

In this study, we demonstrated the antitumor activity of liposomal nanoparticles co-encapsulating ceramides and DXR in in vitro NCL-expressing NB models. It is noteworthy that the comparison between the formulation containing C6-ceramide and DXR and the formulation containing C18-ceramide and DXR revealed a higher antitumor efficacy for the C6-ceramide and DXR nanoparticles both in 2D and multicellular NB tumor spheroid models, as also confirmed by the IC50 values. The enhanced antitumor activity observed with the liposomal formulation co-encapsulating C6-ceramide and DXR may be related to its different membrane-interacting behavior and physicochemical properties compared with C18-ceramide, as suggested by previous reports indicating that short-chain sphingolipids showed superior membrane-interacting properties and consequently improved intracellular delivery capabilities [28].

Multicellular tumor spheroids represent a reliable and cost-effective pre-clinical model, recapitulating the architectural structure of an avascular tumor mass [29,30]. Being structured with a proliferating outer layer, an intermediate layer of quiescent cells, and a necrotic core, they closely mimic the dynamic of oxygen and nutrient exchange. Overall, 3D models, compared to cells cultured in monolayers, are universally recognized as better predictors of a response to therapy. This matter acquires higher relevance in the context of nanocarrier testing, allowing for the evaluation of their ability to penetrate a solid and established structure. The growing number of studies dedicated to this topic demonstrates the great interest of the research community in finding reliable models with predictive value to accelerate clinical translation [29]. Based on this, the antitumor effect achieved by the combination of liposomal ceramide-C6/DXR on both 2D and 3D models of NB increases its preclinical therapeutic value.

A limitation of this study is represented by the lack of in vivo experiments on both cell-line-derived and patient-derived NB models for a clinically translational perspective. Indeed, to support and confirm these data, therapeutic efficacy experiments in clinically relevant animal models of NB [19,31] are necessary. However, in support of the antitumor efficacy of liposomes encapsulating short-chain ceramides, a phase 1 clinical trial has recently been published. In this trial, patients with advanced solid tumors received infusion of liposomes containing C6-ceramide [32] demonstrating a reasonable safety and tolerability profile, with 38% of patients displaying stable disease in four distinct cancer subtypes [32]. Optimal therapeutic performance depends on balancing nanoparticle stability with efficient intracellular drug release. In vivo, long-chain ceramides, such as C18, are known to increase membrane order and rigidity, potentially enhancing liposomal stability and drug retention [33,34]. For this reason, a comparison between the formulation containing C6-ceramide and DXR and the formulation containing C18-ceramide and DXR will also be evaluated in vivo. However, while C18 may improve pharmacokinetic properties, increased bilayer stability can also limit drug release, thereby reducing cytotoxic efficacy compared to short-chain ceramides. This highlights the critical balance between nanoparticle stability and intracellular bioavailability [35,36], and stability and release assays in human plasma and serum-containing media are therefore mandatory.

With this study, we demonstrated that the NCL-targeted C6-ceramide/DXR formulation exerted an enhanced antitumor effectiveness against NB cell lines compared with the formulation bearing only DXR. The results obtained seem to suggest that the NCL-targeted co-administration of DXR and C6-Cer is beneficial against a panel of NB cell lines, irrespective of their *MYCN* status, highlighting the benefit of co-encapsulation. This issue is of particular interest and deserves to be further explored in future studies. It should be noted that empty and ceramide-only liposomal nanoparticles, as well as liposomes functionalized with a scrambled peptide, were not evaluated in the present NB models, and mechanistic investigations are currently lacking. These aspects will be addressed in future studies. Finally, cell-surface NCL expression was here validated on both adrenergic- and mesenchymal-type NB cells. However, the studies conducted and the results obtained so far are insufficient to suggest a possible difference between the two cell subtypes in terms of sensitivity to the proposed liposomal combination therapy.

## 5. Conclusions

Cell-surface nucleolin (NCL) expression was confirmed to play an important role as a promising target in NB, and F3-targeted liposomal nanoparticles were found to selectively deliver their cargo to NCL-expressing neuroblastoma (NB) cells. Interestingly, the combination of liposomal C6-ceramide and doxorubicin against both 2D and 3D models of NB in vitro showed superior antitumor activity compared to liposomal C18-ceramide and doxorubicin and to liposomal doxorubicin alone, supporting ceramide–doxorubicin co-encapsulation as a potential novel treatment strategy for NB and highlighting the benefit of co-encapsulation.

## Figures and Tables

**Table 1:** Z-average particle size, polydispersity index, and zeta potential of liposomal nanoparticles. Ceramide (C6 or C18) and doxorubicin (DXR)-co-encapsulated untargeted (lipo[C6-DXR] and lipo[C18-DXR]) and F3-functionalized (F3-lipo[C6-DXR] and F3-lipo[C18-DXR]) nanoparticles evaluated for size, polydispersity index and zeta potential after production. Results are expressed as the average values ± standard deviation.

Formulation	Mean Particle Size (nm)	Polydispersity Index	Zeta Potential (mV)
lipo[C6-DXR]	72.93 ± 0.47	0.09 ± 0.01	−1.85 ± 0.2
lipo[C18-DXR]	77.95 ± 1.25	0.16 ± 0.03	−1.4 ± 0.3
F3-lipo[C6-DXR]	98.36 ± 1.08	0.18 ± 0.02	+2.55 ± 0.07
F3-lipo[C18-DXR]	101.4 ± 0.9	0.19 ± 0.01	+1.625 ± 0.3

## Data Availability

The raw data supporting the conclusions of this article will be made available by the authors on request.

## Supplementary Materials

The following supporting information can be downloaded at: https://www.mdpi.com/article/10.3390/cells15110958/s1, Figure S1. Antitumor effect of co-encapsulated liposomal nanoparticles of ceramides and doxorubicin on neuroblastoma cells cultured as monolayer.
