# Supplementary material for: Antitumor Activity of Liposomal Nanoparticles Co-Encapsulating Ceramides and Doxorubicin in In Vitro Nucleolin-Expressing Neuroblastoma Models
Source: Cells. 2026 May 22;15(11):958. doi: 10.3390/cells15110958 (PMC13257010; doi:10.3390/cells15110958)
Supplement: Supplementary file 1 [file cells-15-00958-s001.zip › cells-4319439-supplementary.pdf]

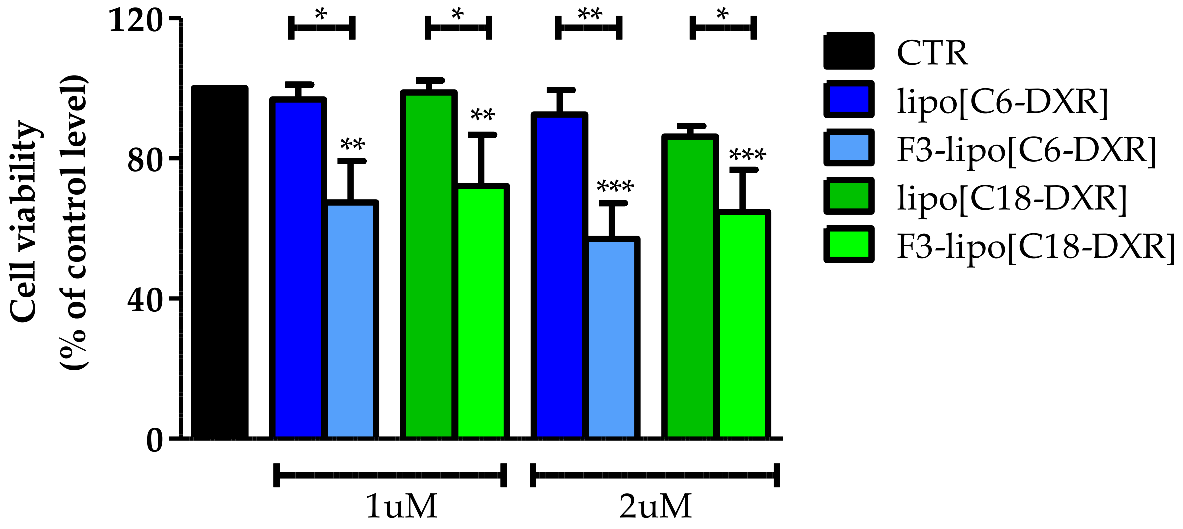

Figure S1. Antitumor effect of co-encapsulated liposomal nanoparticles of ceramides and doxorubicin on neuroblastoma cells cultured as monolayer. Cytotoxicity induced by untargeted (lipo[C6-DXR] and lipo[C18-DXR]) and NCL-recognizing (F3-lipo[C6-DXR] and F3-lipo[C18-DXR]) liposomes on SK-N-AS neuroblastoma cell line. Histograms represent the percentage (%) of cell viability over control (CTR) ± Standard Deviation (S.D). 1 – 2μM: doses of DXR used. Statistics: One- way analyses of variance (ANOVA) with Tukey's Multiple Comparison Test, *, $p < 0.05$; **, $p < 0.01$; ***, $p < 0.001$.
